# Autophagy deficiency exacerbates iron overload induced reactive oxygen species production and apoptotic cell death in skeletal muscle cells

**DOI:** 10.1038/s41419-022-05484-3

**Published:** 2023-04-07

**Authors:** Hye Kyoung Sung, Mayoorey Murugathasan, Ali A. Abdul-Sater, Gary Sweeney

**Affiliations:** 1 Department of Biology, York University, Toronto, ON Canada; 2 School of Kinesiology and Health Science, York University, Toronto, ON Canada

**Keywords:** Cell death, Autophagy

## Abstract

Iron overload is associated with various pathological changes which contribute to metabolic syndrome, many of which have been proposed to occur via damaging tissue through an excessive amount of reactive oxygen species (ROS) production. In this study, we established a model of iron overload in L6 skeletal muscle cells and observed that iron enhanced cytochrome c release from depolarized mitochondria, assayed by immunofluorescent colocalization of cytochrome c with Tom20 and the use of JC-1, respectively. This subsequently elevated apoptosis, determined via use of a caspase-3/7 activatable fluorescent probe and western blotting for cleaved caspase-3. Using CellROX deep red and mBBr, we observed that iron increased generation of reactive oxygen species (ROS), and that pretreatment with the superoxide dismutase mimetic MnTBAP reduced ROS production and attenuated iron-induced intrinsic apoptosis and cell death. Furthermore, using MitoSox Red we observed that iron enhanced mROS and the mitochondria-targeted anti-oxidant SKQ1 reduced iron-induced ROS generation and cell death. Western blotting for LC3-II and P62 levels as well as immunofluorescent detection of autophagy flux with LC3B and P62 co-localization indicated that iron acutely (2–8 h) activated and later (12–24 h) attenuated autophagic flux. We used autophagy-deficient cell models generated by overexpressing a dominant-negative Atg5 mutant or CRISPR-mediated ATG7 knock out to test the functional significance of autophagy and observed that autophagy-deficiency exacerbated iron-induced ROS production and apoptosis. In conclusion, our study showed that high iron levels promoted ROS production, blunted the self-protective autophagy response and led to cell death in L6 skeletal muscle cells.

## Introduction

An often-overlooked feature of the metabolic syndrome is the common occurrence of iron overload, particularly upon aging [1]. Indeed, the term dysmetabolic iron overload syndrome (DIOS) has been coined to describe the association of increased iron levels, characterized by hyperferritinemia with normal or moderately increased transferrin saturation, with various components of metabolic syndrome observed [1]. Excess amounts of iron can impact glucose homeostasis at multiple levels [2]. Whilst the effects of iron on multiple tissues converge to dictate the physiological outcomes, at the core of metabolic dysfunction is the skeletal muscle. Indeed, even in the absence of systemic iron accumulation, muscle iron content increased with aging [3]. Importantly, prolonged iron overload has been shown to attenuate autophagy and induce insulin resistance in skeletal muscle [4]. The effects of excess iron on sarcopenia are remarkably less clear, yet could be of great significance given our growing appreciation of muscle loss in elderly patients with metabolic syndrome [5–7]. Both excess and lack of iron have been associated with sarcopenia, indicating a pivotal role for optimal iron homeostasis in avoiding this deleterious outcome [8, 9].

Whether excess iron levels elicit skeletal muscle damage or atrophy, and how it occurs, requires further investigation. Iron excess in mice led to skeletal muscle atrophy which was reduced by an oxidative stress inhibitor [10]. It was also shown that increased muscle iron levels occurred upon atrophy due to disuse in the aged muscle [11]. Another study indicated that iron played an important role in increasing oxidative stress in muscle atrophied by immobilization, an effect mitigated by iron chelation [12].

A principal cellular mechanism via which excess iron leads to many deleterious effects is the induction of oxidative stress [13]. This can occur via routes such as Fenton chemistry or by promoting excess production from mitochondrial electron transport chain [13, 14]. Accordingly, the use of antioxidants can attenuate the adverse effects of iron excess in various cells and tissues [15]. More recently, the regulation of autophagy flux by iron has been demonstrated in various cells and tissues, although the effects appear to be cell-type specific and highly dependent on the time of iron exposure [4, 16, 17].

In this study we used a cellular model of iron overload (IO) in L6 skeletal muscle cells and determined the consequences of high iron levels on cell death. Mechanistic analysis focused on the role of ROS production, in particular from mitochondria, as well as the significance of autophagy in IO-induced oxidative stress and cell death.

## Materials and methods

### Reagents

The cell culture medium Alpha modification of Eagle’s Medium (AMEM), fetal bovine serum (FBS), and antibiotics/antimycotic solution were purchased from Wisent (St Bruno, QC, Canada). FeCl_3_ (Cat#451649), MnTBAP (Mn (III) tetrakis (4-benzoic acid) porphyrin, Cat#475870), Chloroquine diphosphate salt (Cat#C6628) and Rapamycin (Cat#R8781) were purchased from Sigma‐Aldrich (Ottawa, ON, Canada). Caspase 3 (Cat#9662), LC3B (Cat#2775), GAPDH (Cat#2118) and horseradish peroxidase‐conjugated secondary antibodies (anti‐rabbit‐IgG, Cat# #7074) were purchased from Cell Signaling Technology (Beverly, MA, USA). CellROX Deep Red Reagent (Cat#C10422), CellEvent Caspase 3/7 Green Detection Reagent (Cat#C10423), Alexa 488‐conjugated antibody (goat anti‐rabbit, Cat#A‐11008), Alexa 555‐conjugated antibody (goat anti‐rabbit, Cat#A‐21428) and ProLong Gold (Cat#P36930) were purchased from Thermo Fisher Scientific (Burlington, ON, Canada). Anti‐LC3B (Cat#PM036) and Anti‐p62 (Cat# M162‐3) were purchased from MBL. TOM20 (Cat#sc-17764) was purchased from Santa Cruz Biotechnology, Inc. SkQ1 (Visomitin) [10-(6′-plastoquinonul) decyltriphenylphosphonium] was obtained from Med Chem Express. VECTASHIELD Antifade Mounting Medium with DAPI (Cat# H‐1200) was purchased from Vector Laboratories. Polyvinylidene difluoride membrane was obtained from Bio‐Rad Laboratories, Inc (Burlington, ON, Canada) and chemiluminescence reagent plus was purchased from PerkinElmer (Boston, MA). All other reagents and chemicals used were of the highest purity available.

### Cell culture

L6 skeletal muscle cells were maintained in Alpha modification of Eagle’s Medium (AMEM) from Wisent Bioproduct supplemented with 10% fetal bovine serum (FBS) from Gibco and 1% Antibiotic-antimycotic (vol/vol) from Gibco. Cells were maintained at 37 °C with 5% CO_2_ in a 75 cm^2^ flask. During cell growth, when cells reached 80% confluence, they were trypsinized and seeded for experiments. According to treatment conditions specified in the results, cells were treated with 0.5% FBS AMEM and FeCl_3_ 250 µM and MnTBAP 100 µM. Lentivirus was used to engineer cells overexpressing eGFP‐mCherry‐LC3B L6 and a stable polyclonal population was obtained through puromycin (1 μg/ml) selection for stable integration. Autophagy-deficient L6 cell stably overexpressing dominant negative Atg5 with lysine(K) mutated to arginine(R) at position 130 was generated as described before [18]. CRISPR knock-out of Atg7 was generated as previously described using guide RNAs cloned into pX459 [19]. Vectors carrying guides targeting Atg7 were transfected into L6 cells using Lipofectamine 3000. Transduced cells were selected using 1 μg/ml puromycin over 5 days, and then expanded in media without selection. T7 endonuclease assays were carried out using T7 endonuclease I (NEB) according to the manufacturer’s recommendations on PCR products generated using the following primer pair 5′-GCTGCTGCAGGTAGGTGTAA and 5′- GGTGTCCTGTCTGAGACTGC. Human Skeletal muscle cells were a kind gift from Dr Romel Somwar, Memorial Solan Kettering Cancer Center, New York City, USA. Human skeletal muscle myoblasts obtained from Lonza Bioscience (Hayward, CA) were immortalized by serial transduction with lentiviruses harboring CDK4 (blasticidin) and TERT (hygromycin) cDNAs. Cells stably expressing each construct were selected with the appropriate antibiotics (15 µg/mL blasticidin or 250 µg/mL hygromycin).

### Determination of intracellular iron

Intracellular iron concentration was first estimated by Phen green SK (PGSK) diacetate dye [20] as well as by the endoperoxide reactivity-based FRET probe FIP-1 for ratiometric fluorescence imaging of labile intracellular iron [21]. FIP-1 links two fluorophores (fluorescein and Cy3) through an Fe (II)-cleavable endoperoxide bridge, where Fe (II)-triggered peroxide cleavage leads to a decrease in fluorescence resonance energy transfer (FRET) from the fluorescein donor to Cy3 acceptor. FIP-1 responds to Fe (II) in aqueous buffer with selectivity over competing metal ions and is capable of detecting changes in labile iron pools within living cells. This was provided via a kind gift from Dr Christopher J. Chang, University of California, Berkeley.

### Western blot analysis

After treatment, L6 cells were washed in phosphate buffered saline (PBS). 200 μL of lysis buffer [0.5 M tris-hydrochloride (Tris-HCL) pH 6.8, 2% (vol/vol) sodium dodecyl sulphate (SDS), 15% (vol/vol) glycerol, 10% (vol/vol) 2-mercaptoethanol, and protease inhibitor cocktail] was added to each well and cells were scraped off. The lysates were then incubated at 90 °C for 10 min. Approximately 20 μg of protein was loaded to each well. Samples were analyzed by SDS-polyacrylamide gel electrophoresis (SDS-PAGE) and transferred to a polyvinylidine fluoride (PVDF) membrane. The membranes were blocked in 3% BSA for 1 h and were left in primary antibodies overnight. Western blot analysis was performed using specific primary antibodies to Caspase 3 (Cat#9662), LC3B (Cat#2775) and GAPDH (Cat#2118) (1:1000, Cell Signaling). HRP-linked secondary anti-rabbit (Cat# #7074, 1:5000, Cell Signaling, MA) was used as secondary antibody and proteins were detected by enhanced chemiluminescence reagent (BioRad). The membranes for caspase-3 were stripped each time and re-probed with antibodies against GAPDH (1:1000, Cell Signaling, MA) as loading controls. Band intensity was quantified using ImageJ software.

### Detection of mitochondrial cytochrome c release

L6 cells were grown on glass coverslips in 24-well plate. They were allowed to reached 70% confluency and treated with 0.5% FBS media, containing FeCl_3_ 250 µM with or without the co-treatment of MnTBAP 100 µM for 24 h. At the treatment end point, the coverslips were washed briefly with PBS + + (PBS with added Mg^2+^ and Ca^2+^) and fixed with 10% formalin solution for 30 min. Following 3 more washes, the cells were permeabilized using PBS + + containing 0.5% Triton-X detergent for 30 min and blocked with PBS + + containing 3% BSA for 1 h. Afterwards, the cells were incubated in 3% BSA in PBS + + solution containing rabbit-derived anti-TOM20 (Santa Cruz, for mitochondria detection) and mouse-derived anti-cytochrome c (Santa Cruz) at 1:250 dilution overnight at 4 °C. Cells were then washed 3 times with PBS + + containing 0.5% Tween-20 and incubated in 3% BSA in PBS + + solution containing anti-rabbit IgG Alexa Fluor 555 and anti-mouse IgG Alexa Fluor 488 at 1:800 dilution for 1 h at room temperature. Afterwards, cells were washed 3 times with PBS + + containing 0.5% Tween-20. Coverslips were mounted onto slides in mounting medium containing a 1:1 mixture of VECTASHIELD Mounting medium with DAPI (Vectorlab) and ProLong Gold Antifade Mountant (Thermo Fisher) and imaged using the Nikon A1 Confocal Microscope.

### Immunofluorescence and confocal microscopy

LC3B immunofluorescence was performed as described previously [22]. Briefly, cells were fixed, permeabilized, and blocked with PBS solution containing 1% BSA and 2% goat serum. After blocking, cells were incubated with blocking solution containing LC3B (Cat#PM036, MBL, 1:250). Cells were incubated with anti‐mouse Alexa Fluor 488 secondary antibody (Cat#ab25630, Thermo Fisher Scientific, 1:800) at room temperature for 1 h. After incubation, cells were mounted with DAPI after washes. Deconvoluted images were captured with Nikon A1 confocal microscope. For live cell imaging, L6 cells stably expressing eGFP‐mCherry‐LC3B were seeded into ibidi chambers and treated with FeCl_3_ 250 μM. Images of the whole field were captured in 2D as well as in 3D ( > 100 z- stacks). Images and video acquisitions were performed using a Nikon A1R confocal laser scanning microscope system (Nikon Corp., Tokyo, Japan). Video acquisition at a speed of 30 frames per second was performed for the indicated times, followed by time-lapse imaging every 1 h up to 24 h after iron treatment.

### Detection of ROS using CellROX® Deep Red

For live detection of ROS production, L6 cells were seeded at 70% confluency on a µ-slide 4-well chambered polymer coverslip (Ibidi), and pre-stained 15 min prior to imaging with Hoechst nuclear dye (concentration) (x). The growth medium was then replaced with 0.5% FBS AMEM. At the start of imaging, CellROX® Deep Red at 2.5 µM was added, alongside FeCl_3_ 250 µM in appropriate wells. Imaging was performed at 20x objective (Nikon A1 confocal microscope) and 30 min intervals up to 4 h in a 5% CO_2_ live-cell chamber.

### Caspase 3/7 detection by imaging

CellEvent Caspase 3/7 Detection Reagent is a four-amino acid peptide (DEVD) bound to a nucleic acid-binding dye that is non-fluorescent until cleaved from the peptide (by activated Caspases 3 and 7) and bound to DNA. For detection of apoptotic signal activation, L6 cells were plated at 70% confluency and treated with FeCl_3_ 250 µM (with or without co-treatment with the antioxidant, MnTBAP, at 100 µM for 24 h. At treatment endpoint, Caspase 3/7 Detection Reagent was added to a final concentration of 10 µM and Hoechst to a final concentration of 1 µg/mL, and allowed to incubate for 30 min at 37 °C in the dark. Afterwards, the images were obtained and analyzed to characterize cells with activated Caspase 3/7 pathways through the CellInsight CX7 High Content Signalling platform and Nikon A1 confocal microscope.

### Live/dead and caspase-3 staining by flow cytometry

L6 cells were plated on 6-well plates and cultured to reach 80% confluency. Cells were then treated with 0.5% FBS media, FeCl_3_ 250 µM, or H_2_O_2_ 500 µM with or without co-treatment with MnTBAP 100 µM. At the treatment endpoints, cells were trypsinized, centrifuged and washed 3 times with PBS at 4 °C. Cells were stained in LIVE/DEAD™ Fixable Aqua Dead Cell Stain (1:800; Thermo Fisher) diluted in FACS buffer (DPBS + 2% FBS) for 15 min at 4 °C and washed twice. Cells were then fixed in IC fixation (eBioscience) buffer at RT for 30 min and permeabilized with 100% ice cold methanol at 4 °C for at least 20 min. Caspase-3 antibody (1:50; clone D3E9; Cell Signaling) diluted in FACS buffer was used to stain the cells for 1 hr at RT in 96 well plates (protected from light). Stained cells were then washed twice with FACS buffer and analysed in Attune NxT flow-cytometer (Thermo Fisher).

### Mitosox Red, mBBr and JC-1 assays by flow cytometry

L6 cells were plated on 6-well plates and cultured to reach 80% confluency. Cells were then treated with 0.5% FBS media, FeCl_3_ 250 µM, or H_2_O_2_ 500 µM with or without co-treatment with MnTBAP 100 µM. At the treatment endpoints, cells were trypsinized, centrifuged and washed 3 times with PBS at 4 °C. For oxidative stress stain, cells were stained with 2.5 µM MitoSox Red (Thermo Fisher) and 40 µM mBBr (Thermo Fisher) diluted in FACS buffer for 10 min at 37 °C. For mitochondrial membrane potential stain, cells were stained with 5 µM JC-1 (Thermo Fisher) diluted in FACS buffer for 10 min at 37 °C. Cells were then washed once with FACS buffer and resuspended in FACS buffer and analysed by flow cytometry.

### Annexin V-FITC assay by flow cytometry

L6 cells were plated on 6-well plates and cultured to reach 80% confluency. Cells were then treated with 0.5% FBS media, FeCl_3_ 250 µM, or H_2_O_2_ 500 µM with or without co-treatment with MnTBAP 100 µM. At the treatment endpoints, cells were trypsinized, centrifuged and washed 3 times with PBS at 4 °C. Cells were stained in 1:40 dilution of Annexin V (eBioscience) diluted in 1x binding buffer for 10 min at room temperature. Cells were then washed once in binding buffer and resuspended in 190 μl binding buffer. Finally, 10 μl of PI (1.5 µM) was added to the cells and analysed immediately by flow cytometry.

### LC3B-eGFP-mCherry assay by confocal and flow cytometry

LC3B-eGFP-mCherry L6 cells were plated on 6-well plates and cultured to reach 60% confluency for confocal and 80% confluency for flow cytometry. Cells were then treated with 0.5% FBS media, FeCl_3_ 250 µM or CQ 30 µM or Rapamycin 500 nM. At the treatment endpoints, cells were trypsinized, centrifuged and washed with either PBS containing 0.05% saponin or with PBS alone. Cells were then either imaged by a 20x objective (Nikon A1 confocal microscope) at 1 h intervals up to 24 h in a 5% CO_2_ live-cell chamber or analyzed by flow cytometry (Attune NxT) to assess eGFP fluorescence.

### Lactate dehydrogenase (LDH) assay

LDH assay (#786-210, G-Biosciences) was used to determine cellular cytotoxicity of human skeletal muscle and L6 cells with the treatments indicated in the experiment. LDH is a stable enzyme which is rapidly released into the culture medium upon damage of the plasma membrane. LDH released from the cell oxidizes lactate to generate NADH, which then reacts with water soluble tetrazolium salt (WST) to generate a yellow color. The intensity of the generated colour correlates directly with the number of lysed cells. After the treatment of FeCl_3_ as indicated in both cells, 25 µL of culture media was added to new 96-well plate followed by the addition of 25 µL of reaction mix from the LDH kit. The reaction mix was made accordingly to kit instructions. The plate was incubated for 20 min at 37 °C, 5% CO_2_ and covered with aluminium foil. Then, the cells were measured by a colorimetric assay at wavelengths 490 nm and 680 nm. The values were calculated by subtraction of 680 nm from 490 nm values and normalization by the control in order to reach the final cellular cytotoxicity measurement.

### Assay using ReadyProbe™ cell viability

ReadyProbes assay (#R37609, Invitrogen) was used to determine cell viability of human skeletal muscle cells with treatments indicated in experiments. The ReadyProbe contains the NucBlue® Live reagent, which stains nuclei of all cells. Also, it contains the NucGreen® Dead reagent, which stains only the nuclei of dead cells that have compromised plasma membranes. After adding 1 drop of each reagent for each 1 mL of culture media, the plate was covered with aluminium foil and incubated for 30 min at 37 °C, 5% CO_2_. After incubation, cells were detected with a standard DAPI (blue) and GFP (green) filters using EVOS microscopy. The data were analyzed using ImageJ software, then the % of dead cells calculated by the ratio between number of dead cells to the total number of cells.

### Statistical analysis

All data are presented as mean ± SEM. Statistical analysis was performed using Student’s t-test when comparing 2 groups. To compare experiments with more than 2 groups, one-way ANOVA followed by Dunnett’s post-test was performed. The differences among groups were considered statistically significant when *p* < 0.05 and are indicated by symbols as described in figure legends.

## Results

### Intracellular iron accumulation and cell death in skeletal muscle cells

**Fig. 1:**
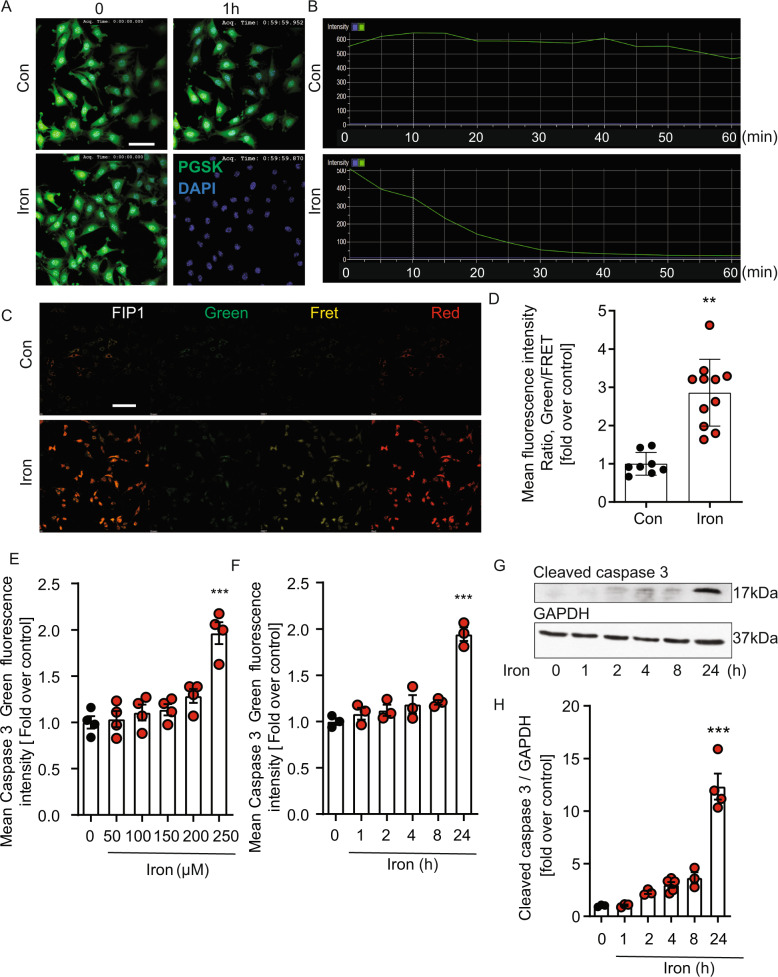
Validation of a cellular model of intracellular iron accumulation and induction of cell death. Time-lapse images examining intracellular iron level in L6 skeletal muscle cells, using PGSK. Cells were treated with 250 µM FeCl_3_ for up to 60 min concurrently with PGSK dye. **A** Representative images taken at time 0 and 60 min without or with FeCl_3_ treatment and **B** quantification of PGSK mean fluorescence intensity from images taken every 5 min (*n* = 3). **C** shows representative images obtained using FRET iron probe 1 (FIP‐1) without or with FeCl_3_, 250 μM for 24 h and quantification of FIP-1 (Green/FRET ratio) is shown in **D** (*n* = 3). Quantification of caspase 3/7 activity was assessed using an activatable fluorescent substrate done by CellInsight CX7 to examine dose- and time-dependent apoptotic cell death with a dose‐response to FeCl_3_ (50, 100, 150, 200 and 250 µM) shown in **E** and at multiple time points (250 µM FeCl_3_, at 1, 2, 4, 8, 24 h) in **F** (*n* = 3). To verify these observations, western blot analysis of cleaved caspase 3 was determined after treatment of cells with 250 µM FeCl_3_, at 1, 2, 4, 8, 24 h **G** and quantification is shown in **H** (*n* = 5). Scale bar in A 50 µm and C 100 µm. All graphs show mean ± SEM and * = *P* < 0.05, ** = *P* < 0.01, *** = *P* < 0.001 versus control.

To monitor cellular iron uptake, both an iron responsive fluorophore and a ratiometric probe were used to assess the intracellular content of labile iron. L6 cells were pretreated with the fluorophore Phen Green SK (PGSK), which is quenched by iron, 30 min before iron treatment followed by real-time kinetic analysis of fluorescence every 5 min for 1 h. The change in fluorescent signal intensity was compared to controls cultured for the same time period without iron treatment and a clear quenching of the fluorescence was visible in cells treated with iron (Fig. 1A, B). In addition, the FRET probe (FIP1) was used to confirm the iron excess in L6 cells treated with 250 μM FeCl_3_ for 24 hr (Fig. 1C, D). The mean fluorescent intensity (MFI) ratio of fluorescein (green) over FRET (yellow) increased significantly with iron treatment. Thus, collectively both assays showed a time-dependent increase in intracellular iron accumulation. The iron responsive fluorophore probe Phen Green SK (PGSK) was also added to human skeletal muscle cells, and time lapse analysis up to 6 hr of iron treatment (Supplementary Fig. 1A, B) showed a time-dependent increase in intracellular iron accumulation. To examine whether iron overload causes apoptosis, cells were treated with up to FeCl_3_ 250 µM for up to 24 h and activation of caspase3/7 activity was detected first by using an indicator which becomes fluorescent upon cleavage by active caspase-3/7. Our data demonstrated dose- and time-dependent activation of caspase-3/7 by iron with a significant increase observed after 24 h with 250 µM FeCl_3_ (Fig. 1E, F). Additionally, a significant time-dependent increase of cleaved caspase-3 detected by western blot was observed upon iron treatment (Fig. 1G, H). To examine whether iron overload causes apoptosis in human skeletal muscle cells, we used LDH release detection assay (Supplementary Fig. 1F) and apoptotic cells with compromised plasma membrane detected by image-it DEAD kit (Supplementary Fig. 2G, H) for measuring cell death. The data indicated that there was a significant increase in cell death after 24 h of iron treatment.

### Iron induced ROS production in skeletal muscle cells

**Fig. 2:**
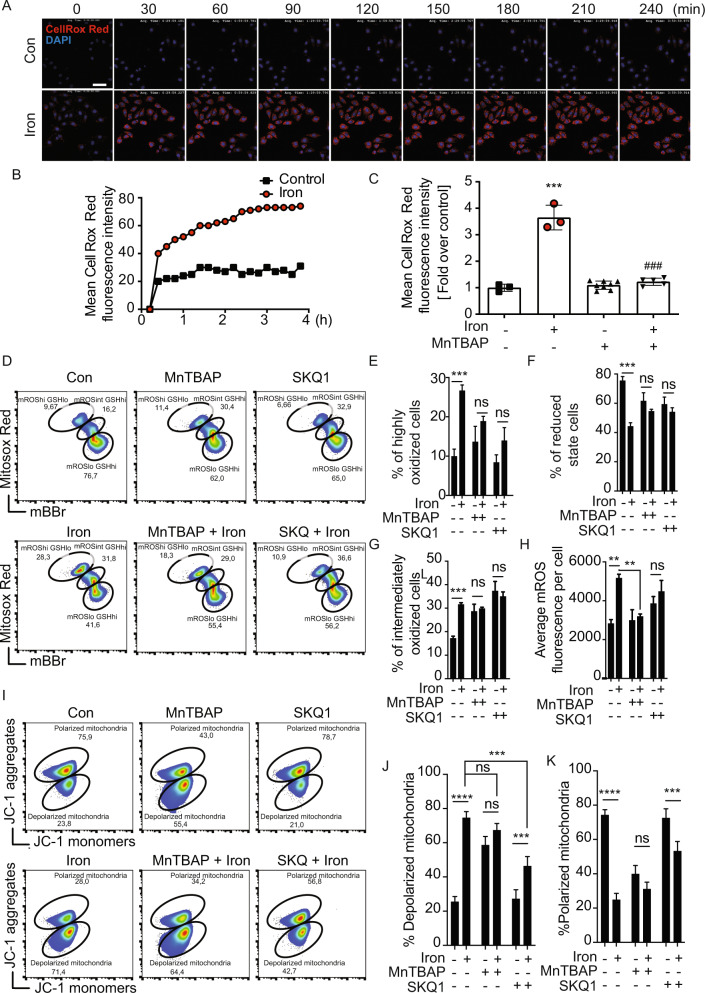
Examination of iron-induced ROS production. Using CellROX Deep Red to detect ROS we performed confocal microscopy analysis with time-lapse acquisition at 30 min intervals up to 4 h **A** in the presence or absence of iron (250 µM FeCl_3_) with quantitation shown in **B** (*n* = 3). The effect of anti-oxidant 100 µM MnTBAP for 30 min prior to iron treatment on iron-induced ROS production was assessed using CellROX Deep Red after treatment of cells with 250 µM FeCl_3_ for 4 h by confocal microscopy **C** (*n* = 3). **D** Representative plots from one experiment showing frequency of cell populations in oxidized (mROShi GSHlo), intermediate (mROSint GSHhi) and reduced (mROSlo GSHhi) states based on intracellular levels of glutathione (GSH; measured by mBBr) and mitochondrial ROS levels (mROS; measured by MitoSox Red) upon treating cells with 250 µM FeCl_3_ for 4 h (Iron) in the presence or absence of 100 µM MnTBAP or 20 nM SKQ1. Graphs show percentages of cells in highly oxidized **E**, reduced **F**, and intermediately oxidized **G** states from three experiments as in **D**. **H** Graphs show the average mROS fluorescence per cell from three experiments as in **D** (*n* = 3). **I** Representative plots showing frequency of cell populations with depolarized (JC-1 monomers) and polarized mitochondria (JC-1 aggregates) upon treating with 250 µM FeCl_3_ for 4 h (Iron) in the presence or absence of 100 µM MnTBAP or 20 nM SKQ1. Graphs show percentages of cell populations with depolarized **J** and polarized **K** from three experiments as in **I** (*n* = 3). Scale bar denotes 50 µm. All graphs show mean ± SEM and * = *P* < 0.05, ** = *P* < 0.01, *** = *P* < 0.001 versus control, # = *P* < 0.05, ## = *P* < 0.01, ### = *P* < 0.001 versus iron.

We next investigated the effect of iron on intracellular reactive oxygen species (ROS) production. This was first performed using the ROS indicator dye CellROX^®^ red. Representative images from temporal study using live cell imaging are shown in Fig. 2A and quantitative analysis (Fig. 2B) clearly indicated that ROS production reached a maximum within 4 hr and plateaued thereafter. Further quantitative analysis using the same assay indicated a significant increase 4 h after iron treatment and that it could be significantly attenuated by MnTBAP, a superoxide dismutate (SOD) minetic (Fig. 2C). We then further investigated ROS generation using Mitosox Red and mBBR via flow cytometry (Fig. 2D–H). We show that iron significantly increased the number of highly oxidized cells (Fig. 2D, E) and moderately oxidized cells (Fig. 2D, G), while decreasing the number of cells in a reduced state (Fig. 2D, F). Overall mitochondrial ROS (mROS) was similarly increased following iron treatments (Fig. 2H). These effects were abolished by the use of MnTBAP or SKQ1, the latter a mitochondria targeted antioxidant. We also investigated the effect of iron on intracellular ROS production in human skeletal muscle cells using CellRox and on cellular glutathione status by detecting mBBR via flow cytometry (Supplementary Fig. 1C–E) and observed similar alterations as were seen in L6 cells. We show that iron significantly decreased glutathione (GSH) level in human skeletal cells (Supplementary Fig. 1E). Importantly, using JC-1 probe we observed that iron triggered mitochondrial depolarization (Fig. 2I), as evident by the significant increase in cells with unhealthy or depolarized mitochondria (Fig. 2J) and decrease in cells with healthy or polarized mitochondria (Fig. 2K) following iron treatments. SKQ1 significantly but partially reversed iron induced mitochondrial depolarization, suggesting additional mechanisms through which iron disrupts mitochondrial membrane potential.

### Inhibition of iron-induced ROS production attenuates cell death

**Fig. 3:**
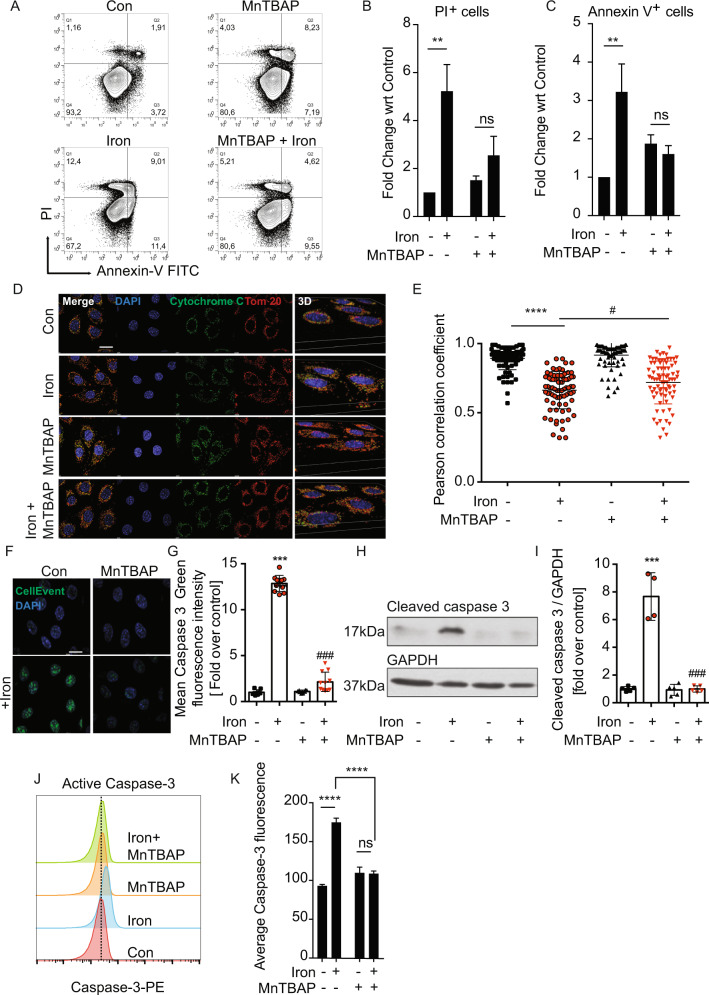
Analysis of the role of ROS in iron-induced apoptotic cell death. **A** Representative plots showing frequency of Annexin-V and/or propidium iodide (PI) labeled cells following treatment with 250 µM FeCl_3_ for 48 h (Iron) in the presence or absence of 100 µM MnTBAP that was added 30 min prior to iron treatment. **B**, **C** Graphs show percentages of Annexin-V positive and PI positive cells from three experiments as in **A** (*n* = 3). Representative immunofluorescence images showing cytochrome c (green), Tom-20/mitochondria (red), nucleus (blue) in cells treated with 250 µM FeCl_3_ for 24 h and 100 µM MnTBAP for 30 min prior to iron treatment **D** (*n* = 3). Merged images are shown in left side column and 3-dimensional rendering in right side column. Colocalization of red/green was quantified and shown in (E) as Pearson’s correlation coefficient. Representative images from caspase 3/7 detection fluorescence assay in cells treated with 250 µM FeCl_3_ for 24 h and 100 µM MnTBAP for 30 min prior to iron treatment and with quantification **F**, **G** (*n* = 3). Western blot analysis of cleaved caspase 3 was performed under similar conditions and a representative image is shown in (H) with quantification of the change in cleaved caspase 3 relative to GAPDH in **I** (*n* = 5). **J** Overlayed histograms showing the mean fluorescence intensity (MFI) of caspase-3 upon treating cells with 250 µM FeCl_3_ for 24 h (Iron) in the presence or absence of 100 µM MnTBAP that was added 30 min prior to iron treatment. **K** Graphs show MFI of caspase-3 from three experiments as in **I** (*n* = 3). Scale bar denotes 20 µm. All graphs show mean ± SEM and * = *P* < 0.05, ** = *P* < 0.01, *** = *P* < 0.001 versus control, #=*P* < 0.05, ##=*P* < 0.01, ###=*P* < 0.001 versus iron. B graph *P* = 0.12, C graph *P* = 0.07 versus iron.

To further investigate the mechanism through which iron overload may induce cell death, we employed the Annexin V/PI assay using flow cytometry. In early apoptosis, phosphatidylserine (PS) is externalized to the outer membrane of the phospholipid bilayer, which can be measured by Annexin V staining. 24 h following incubation with iron, a significant increase in apoptotic cells was observed as shown by FITC-annexin V binding (Fig. 3A, C). Cells were counterstained with propidium iodide (PI) to distinguish between apoptotic and necrotic cells. Our results indicate an increase in both apoptotic (Fig. 3A; Q2 & Q3) and necrotic (Fig. 3A; Q1) cell death. As expected, pretreatment of L6 cells with MnTBAP attenuated the overall increase in Annexin V/PI staining upon iron treatment and protected the cells from iron induced cell death (Fig. 3A–C).

To further investigate the activation of intrinsic apoptosis, a cytochrome C/Tom20 co-localization assay was performed. This assay is designed to determine whether cytochrome C has been released out of mitochondria to the cytoplasm to further activate the cascade reaction of apoptosis. Representative merged images of the two different conditions showed that in control, cytochrome C primarily resided inside mitochondria (represented by yellow puncta (Fig. 3D)). There was a significant decrease in cytochrome C and Tom20 colocalization upon iron treatment. Multiple datasets were then quantified and colocalization was represented as a Pearson’s coefficient ratio (Fig. 3E) which showed the significant cytochrome C release by iron and significant attenuation by MnTBAP. A significant increase in caspase-3 activity was detected after 24 h of treatment with iron by using fluorescent confocal imaging of the caspase-3/7 activatable probe (Fig. 3F, G) and western blotting (Fig. 3H, I). Both sets of data verified inhibition of this process by MnTBAP. Similarly, flow cytometric measurements showed that iron induced an increase in active casapase-3, which was abrogated following the addition of MnTBAP (Fig. 3J, K).

### Iron inhibited autophagy in L6 skeletal muscle cells

**Fig. 4:**
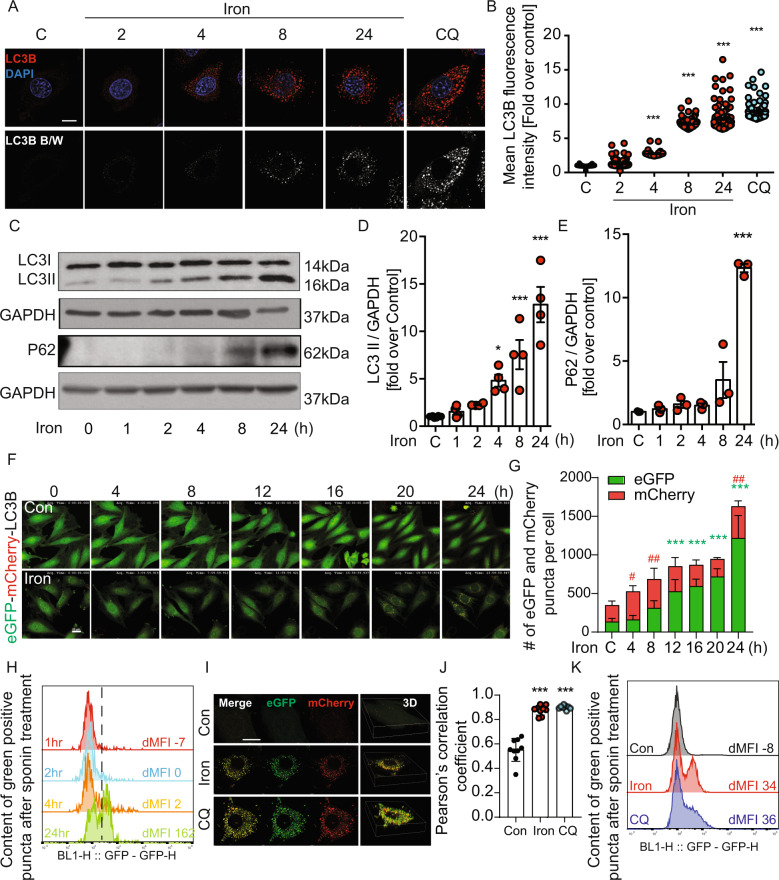
Examination on iron-induced alterations in autophagy flux. Representative immunofluorescent images detecting LC3B in response to 250 µM FeCl_3_, at 2, 4, 8, 24 h or CQ (30 µM, 24 h) and quantification of their mean fluorescent intensity **A**, **B** (*n* = 3). Representative western blotting images of LC3B and P62 **C**, and quantitation relative to GAPDH **D**, **E** after 250 µM FeCl_3_ for 1, 2, 4, 8, 24 h (*n* = 4). Representative confocal microscopy time lapse image of L6 cells stably expressing tandem fluorescent eGFP-mCherry-LC3B after treatment with 250 µM FeCl_3_ **F**. Quantification of eGFP-mCherry-LC3B puncta **G** (*n* = 3). eGFP fluorescence was measured by flow cytometry in L6 cells stably expressing tandem fluorescent eGFP-mCherry-LC3B treated with with 250 μM FeCl_3_ for 1, 2, 4 or 24 h after saponin permeabilization and cytoplasmic washout of the cells. eGFP dMFIs were quantified by subtracting the MFI of WT L6 cells from that of eGFP-mCherry-LC3B cells **H** (*n* = 3). Representative confocal image of L6 cells stably expressing tandem fluorescent eGFP-mCherry-LC3B after treatment FeCl_3_ (250 μM, 24 h) or CQ (30 µM, 24 h) **I**, Pearson’s correlation coefficient **J**, flow cytometer **K** (*n* = 3). Scale bar denotes 20 µm. All graphs show mean ± SEM and * = *P* < 0.05, ** = *P* < 0.01, *** = *P* < 0.001 versus control, #=*P* < 0.05, ##=*P* < 0.01, ###=*P* < 0.001 versus iron.

We next examined the potential mechanistic role of cellular autophagy in determining the effects of iron overload. We first confirmed via temporal analysis using immunostaining for endogenous LC3 puncta (Fig. 4A, B) and western blotting of LC3-II (Fig. 4C, D) that short term iron treatment (4 h, 8 h) increased the number of autophagosome puncta and LC3-II levels. However, at 24 h these changes were accompanied by P62 accumulation (Fig. 4C, E) indicating a shift to inhibition of autophagic flux. To further analyze autophagy flux, we used L6 cells stably expressing fluorescent eGFP-mCherry-LC3B and conducted live time-lapse imaging every 1 hr up to 24 h after iron treatment. In this assay eGFP fluorescence is quenched by low pH after autophagosome fusion with the lysosome, whereas mCherry signal is retained [23]. Using this approach we again observed increased autophagosome content at 4 and 8 h and a significantly increased size and number of eGFP fluorescence puncta after 12–24 h iron treatment (Fig. 4F, G), the latter increased autophagosome to autophagolysosome ratio indicating inhibition of flux. To lend further weight to this conclusion we also used the quantitative power of flow cytometry, which has recently been used to measure autophagy [24]. Saponin-based extraction of eGFP-LC3-I allows the quantification of intracellular eGFP-LC3-II levels, hence this assay can quantify the decrease in green fluorescence as an index of degradation of eGFP-LC3 following its association with autophagosomes. First, we validated this assay by comparing total GFP fluorescence in wildtype L6 (WT L6) and eGFP-mCherry-LC3B L6 cells by flow cytometry (Supplementary Fig. 2A). We specifically measured GFP fluorescence from the autophagosome associated LC3I by selectively extracting the non-autophagosome associated LC3I with 0.05% saponin by flow cytometry (Supplementary Fig. 2B) and confocal microscopy (Supplementary Fig. 2C). Rapamycin and chloroquine (CQ) were used as controls to confirm the specificity of the assay (Supplementary Fig. 2D, E). We next employed this assay to quantify autophagy following iron treatment of eGFP-mCherry-LC3B L6 cells. Remarkably, we showed that autophagosome associated LC3 fluorescence peaked at 24 h following iron treatment (Fig. 4H). We then analyzed the effect of 24 h iron and CQ treatment on autophagosome production and fusion with lysosomes by confocal imaging in L6 cells stably expressing LC3B-eGFP-mCherry. At 24 h after iron and CQ treatment, an increased Pearson correlation coefficient was observed (Fig. 4I, J), indicating a lack of autophagy flux despite the presence of a large pool of autophagosomes. The potency of iron induced autophagosome accumulation was comparable to the effect of chloroquine treatment (Fig. 4K). Thus, results consistently obtained by western blot, confocal microscopy and flow cytometry showed an initial activation (2–8 h) of autophagy and later (12–24 h) reduction in flux upon iron treatment.

### Enhanced iron induced ROS in autophagy deficient cells

**Fig. 5:**
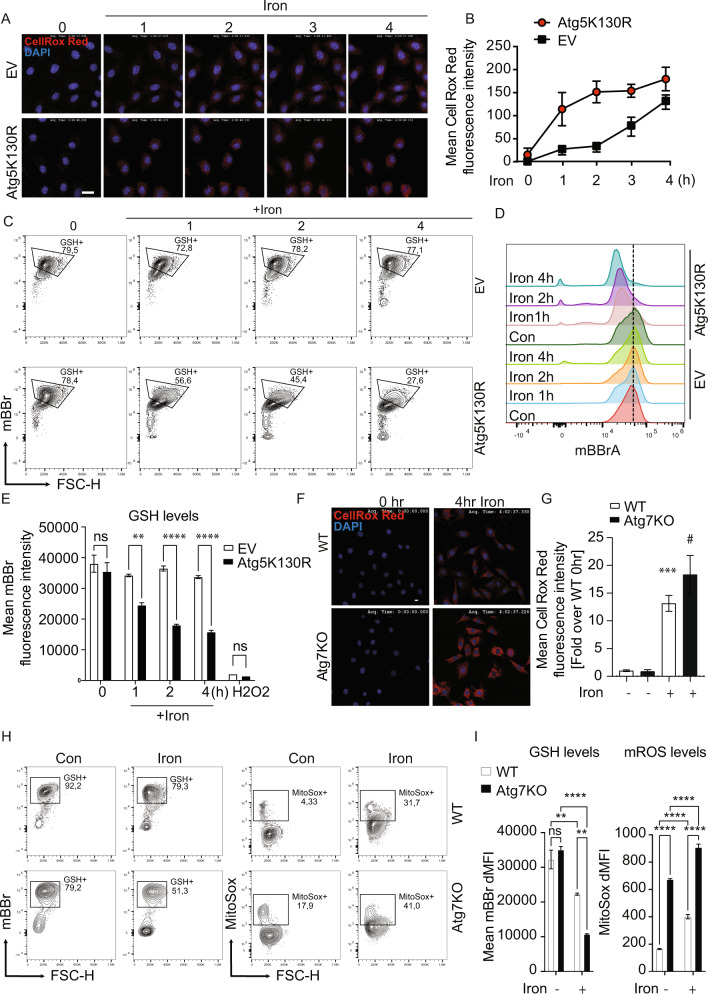
Autophagy deficiency exacerbates iron-induced ROS. CellROX Deep Red was used **A**, **B** to examine ROS production in normal (EV) and autophagy deficient (Atg5K130R) cells. Time-lapse image acquisition at 1 h intervals up to 4 h in cells treated with 250 µM FeCl_3_ **A** with quantitation **B** (*n* = 3). **C** Representative plots from one experiment showing frequency of mBBR labeled cells (GSH + ) upon treatment with 250 µM FeCl_3_ for 1, 2 or 4 h in EV and Atg5K130R cells. **D** Overlayed histograms showing the mean fluorescence intensity (MFI) of mBBR in EV and Atg5K130R cells, and **E** the average mBBr MFI from three experiments as in **C** (*n* = 3). Representative images from CellROX Deep Red fluorescence assay to examine ROS production in WT and autophagy knock out (Atg7KO) cells and 4 h in cells treated with 250 µM FeCl_3_ **F** with quantitation **G** (*n* = 3). Representative plots from one experiment showing frequency of mBBR labeled cells (GSH + ) MitoSox Red upon treatment with 250 µM FeCl_3_ for 4 h in WT and Atg7KO cells. **H** Representative plots from one experiment showing frequency of mBBR labeled cells (GSH + ) and MitoSox labeled cells (MitoSox + ) in WT and Atg7KO cells and **I** the average mBBr and MitoSox Red MFI from three experiments as in **H** (*n* = 3). Scale bar denotes 50 µm for **A** and 10 µm for **F**. All graphs show mean ± SEM and * = *P* < 0.05, ** = *P* < 0.01, *** = *P* < 0.001 versus control, # = *P* < 0.05, ## = *P* < 0.01, ### = *P* < 0.001 versus iron.

To test the functional significance of autophagy, we generated two cellular models of autophagy deficiency. Firstly, a stable cell line with autophagy deficiency induced by overexpression of a dominant-negative Atg5 mutant Atg5K130R was used. Temporal analysis of total cellular ROS production detected using CellROX red by confocal microscope indicated a faster and greater increase in autophagy deficient versus control cells transfected with empty vector (Fig. 5A, B). A similar observation was made upon analysis of mBBr staining by flow cytometry (Fig. 5C–E). To verify the phenomenon of enhanced ROS generation in response to iron in an autophagy deficient setting, we then used CRISPR/Cas9 technology to generate Atg7 knock out cells. Iron significantly increased intracellular ROS production (Fig. 5F, G), reduced GSH level (Fig. 5H, I) and enhanced mROS production (Fig. 5H, I) in WT and ATG7KO cells.

### Autophagy deficiency exacerbates iron-induced apoptosis

**Fig. 6:**
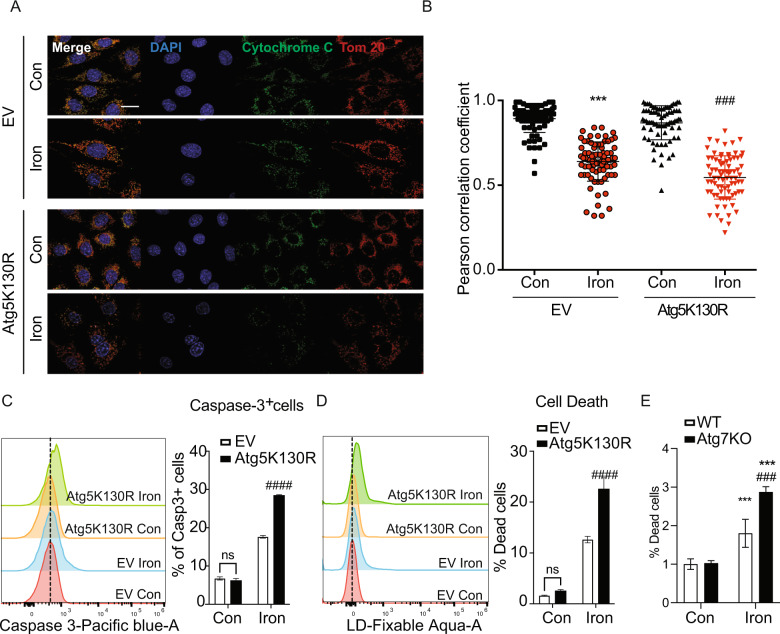
Iron-induced apoptosis is enhanced in autophagy-deficient cells. **A** Representative immunofluorescence images showing cytochrome c (green), Tom-20/mitochondria (red), nucleus (blue) in EV or Atg5K130R cells treated with 250 µM FeCl_3_ for 24 h. Colocalization of red/green was quantified and shown in **B** as Pearson’s correlation coefficient (*n* = 3). **C** Overlayed histograms (left panel) showing the mean fluorescence intensity (MFI) of Caspase-3 in EV and Atg5K130R cells cells upon treating with 250 µM FeCl_3_ for 24 h (Iron), while the graphs (right panel) showing the average of Caspase-3+ cells from three experiments. **D** Overlayed histograms (left panel) showing the MFI dead cells in EV and Atg5K130R cells as in **C**, while the graphs (right panel) show MFI of dead cells from three experiments (*n* = 3). LDH release detection assay for cell death, quantified as 490 nm absorbance in WT and Atg7KO cells **E** (*n* = 3). Scale bar denotes 20 µm. All graphs show mean ± SEM and * = *P* < 0.05, ** = *P* < 0.01, *** = *P* < 0.001 versus control, # = *P* < 0.05, ## = *P* < 0.01, ### = *P* < 0.001 versus WT iron.

To further investigate iron induced apoptosis occurring downstream of increased ROS production in an autophagy deficient setting, we first performed a similar set of apoptosis analysis as previously described, including cytochrome C/Tom20 immunofluorescence colocalization assay and flow cytometry using caspase-3/7 activatable probe and live/dead staining in empty vector and ATG5K130R cells. In both empty vector and ATG5K130R cells, a significant decrease in cytochrome C and Tom20 colocalization with iron treatment was observed (Fig. 6A, B). The fold increase between control and iron-treated groups was greater in ATG5K130R cells. There was a significant increase in the magnitude of iron-induced caspase-3 activation and dead cells detected by flow cytometry in ATG5K130R cells (Fig. 6C, D). Similarly, live/dead assay data also showed that iron induced cell death was exaggerated in Atg7 knockout cells versus normal cells (Fig. 6E). Thus, data from both cellular models of autophagy-deficiency indicated the ability of autophagy to significantly reduce iron-induced apoptosis.

## Discussion

In this study we tested the impact of excess intracellular iron levels on ROS production and cell death in skeletal muscle cells of both rat and human origin. Furthermore, we focused on the mechanistic role of autophagy in determining the extent of cell death in response to iron. We have previously shown that treating L6 cells with media containing FeSO_4_ led to accumulation of intracellular iron and here we used both the fluorescent heavy metal indicator PGSK as well as the endoperoxide reactivity-based FRET probe FIP-1 [21] with ratiometric fluorescence imaging to confirm time-dependent significant increase in labile iron when cells were treated with FeCl_3_. At the same time, we assessed apoptosis using a caspase-3 activatable fluorescent probe as well as caspase-3 western blotting and observed a time- and dose-dependent increase in response to iron. Characterization of this cellular model then allowed us to investigate mechanisms of iron-induced cell death in skeletal muscle cells.

First, we focused on the role of oxidative stress since it is well established that iron can induce ROS generation via several routes [13]. Since iron can easily switch between the Fe^2+^ and Fe^3+^ forms, providing or accepting electrons, respectively, it is a major player in generation of reactive oxygen species. Mechanisms have evolved to counteract the harmful effects of excess iron, including binding by transferrin or ferritin [25], however when these systems become overloaded and labile iron exists then toxicity can ensue. Labile iron has been shown to elevate ROS levels via Fenton chemistry and cellular sources such as xanthine oxidase, NADPH oxidase and mitochondria. Here, we focused on the latter and found that in the model used, mitochondria were an important source of ROS in response to iron. Mitochondrial respiration and intracellular iron, and in particular intramitochondrial iron, have an intimate association since optimal iron balance is critical for efficient metabolism [26]. During respiration a small amount of O_2_ undergoes partial reduction in the electron transport chain process and this can lead to formation of potentially harmful intermediates, collectively called reactive oxygen species (ROS). These include hydrogen peroxide (H_2_O_2_), superoxide anion (O_2•_^−^) and hydroxyl radicals (HO_•_) [13]. We used both a generic antioxidant MnTBAP as well as the mitochondrial-targeted antioxidant SKQ1 to show that mitochondria were an important source of ROS, measured using CellRox and Mitosox, in response to iron. SKQ1 has shown beneficial effects in many disease states [27–29] and our data suggests that some cases of iron overload induced cellular damage can also benefit from use of this agent.

Previous literature provides a strong rationale for us to hypothesize that an iron to ROS to cell death axis exists in this cellular model. Increased cell death in response to elevated intracellular ROS levels has been shown in a wide variety of cell types and tissues over many years [30]. Indeed, various studies have shown that iron induced ROS leads to cell death [11, 31–33]. The importance of optimal iron levels should be emphasized as too little iron can often elicit similar outcomes [34]. In this study, having proven that MnTBAP and SKQ1 could attenuate iron-induced ROS production and mitochondrial membrane depolarization, we next showed that iron-induced cell death was also attenuated by antioxidant pretreatment. This was established using immunofluorescence to detect cytochrome c release from mitochondria, caspase-3/7 activation by fluorescent microscopy, flow cytometry and western blotting as well as annexin-V/PI analysis using flow cytometry. The results led us to the conclusion that iron-induced ROS production is an important mechanistic step leading to apoptotic cell death.

A main focus of this study was to investigate the regulation of autophagy by iron and its interaction with the iron-ROS-cell death axis [35]. Previously, we showed that in L6 skeletal muscle cells excess levels of FeSO_4_ induced a transient increase in autophagy, followed by a later inhibition of autophagic flux [4]. Here we used fluorescent and immunofluorescent detection of LC3 and P62 colocalization as well as western blotting for both proteins and made a similar conclusion. Other studies have shown similar observations with enhanced autophagy detected initially in response to iron in osteoblast cells [11] and that prolonged iron will suppress autophagy flux in neuroblastoma cells [36], mouse testes [37] and rat hippocampus [17]. It is likely that the cell boosts autophagy in response to iron as a protective compensatory mechanism [38]. However, in keeping with previous studies [4, 17] we found that iron overload overcomes this and is shown to turn down autophagy flux after 24 h, likely via inhibition of autophagosome lysosome reformation (ALR) [4]. This is likely to contribute to the outcome of increased apoptotic cell death since others have shown that autophagy is an important cellular protection mechanism against cell death in response to oxidative and other stresses. For example, epigallocatechin-3-gallate reduced liver damage induced by high fat diet and oxidative stress via increasing autophagy and decreasing apoptosis [39] and inhibition of autophagy in breast cancer cells leads to enhanced proscillardin A-induced apoptosis [40]. Indeed, an important conclusion from this study was that iron had exaggerated effects on ROS production and cell death in autophagy deficient cells generated either by overexpressing the dominant-negative Atg5K mutant or by CRISPR-mediated deletion of Atg7.

In summary, our data indicates that iron-induced ROS production plays a critical role in the induction of mitochondrial-dependent apoptotic cell death. Whereas the cell responds to this perturbation via attempting to induce autophagy and resolve optimal homeostasis and function, iron also acts to suppress autophagy flux and thus leads to enhanced cell death after chronic treatment. These observations in a cellular skeletal muscle model could have important translational significance since high levels of iron are known to be associated with muscle wasting [10, 41] and reduced muscle regeneration upon aging [42].

## Supplementary information

Figure 1

Figure 2

Supp Fig Legends

Original Data File

## Data Availability

The data that support the findings of this study are available from the corresponding author GS upon reasonable request.
