# Supplementary figures and images for: Autophagy deficiency exacerbates iron overload induced reactive oxygen species production and apoptotic cell death in skeletal muscle cells
Source: Cell Death Dis. 2023 Apr 7;14(4):252. doi: 10.1038/s41419-022-05484-3 (PMC10081999; doi:10.1038/s41419-022-05484-3)

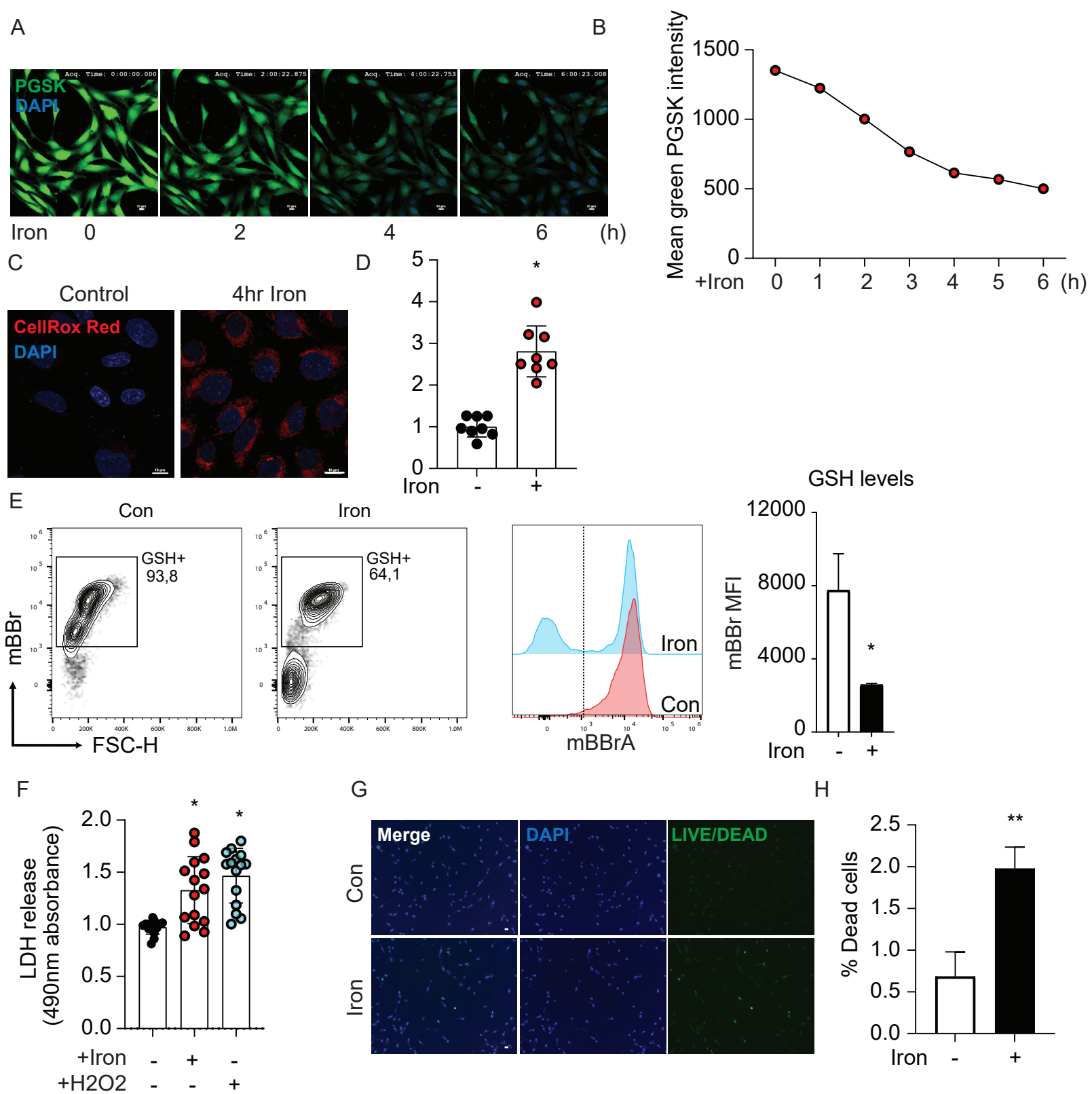

Supplement: Supplementary file 1 — Figure 1 [file 41419_2022_5484_MOESM1_ESM.pdf]

A

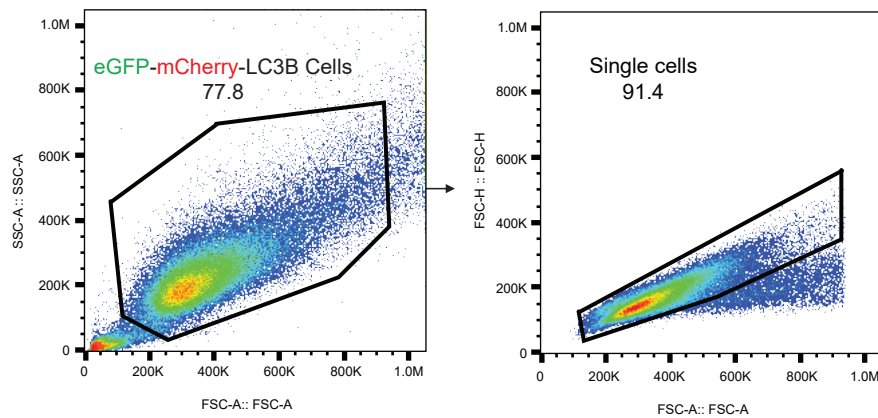

WT

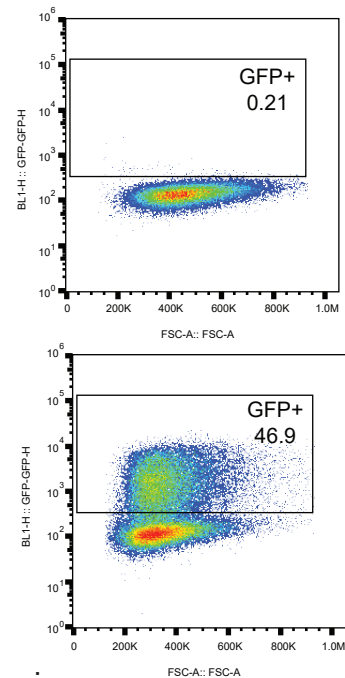

eGFP-mCherry-LC3B

B

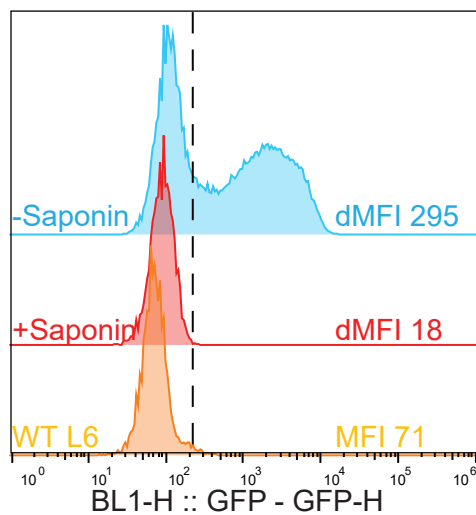

C

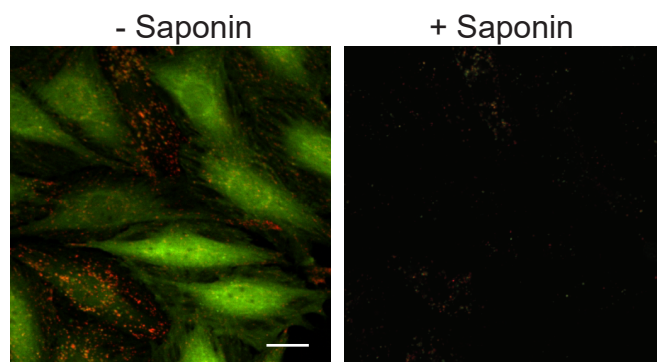

D

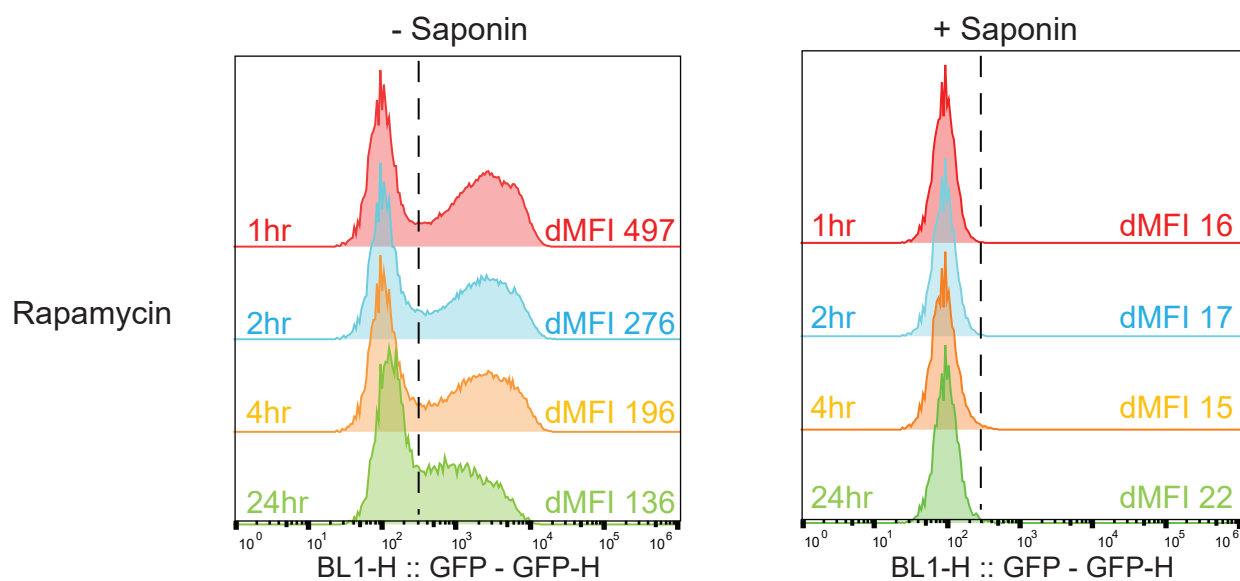

E

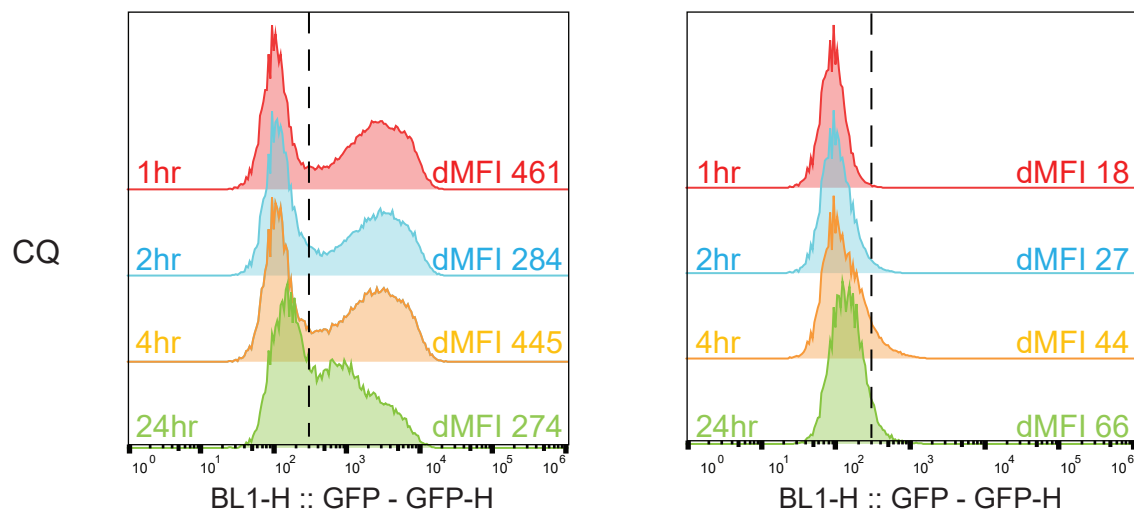

Supplement: Supplementary file 2 — Figure 2 [file 41419_2022_5484_MOESM2_ESM.pdf]
