# Supplementary material for: Autophagy deficiency exacerbates iron overload induced reactive oxygen species production and apoptotic cell death in skeletal muscle cells
Source: Cell Death Dis. 2023 Apr 7;14(4):252. doi: 10.1038/s41419-022-05484-3 (PMC10081999; doi:10.1038/s41419-022-05484-3)
Supplement: Supplementary file 3 — Supp Fig Legends [file 41419_2022_5484_MOESM3_ESM.docx]

**Supplementary Figure 1. Examination of key assays in Human skeletal muscle cells.**

Time-lapse images examining intracellular iron level in Human skeletal muscle cells, using PGSK. Cells were treated with 1mM FeCl_3_ for up to 6h concurrently with PGSK dye. (A) shows representative images taken at time 0 and 6h with FeCl_3_ treatment and (B) quantification of PGSK mean fluorescence intensity from images taken every 1h (n=3). Using CellROX Deep Red to detect ROS we performed confocal microscopy analysis at 4h (C) in presence or absence of iron (1mM FeCl_3_) with quantitation shown in (D) (n=3). Representative plots from one experiment showing frequency of mBBR labeled cells (GSH+) upon treatment with 1mM FeCl_3_ for 4h in Human skeletal muscle cells. (E) Overlayed histograms showing the mean fluorescence intensity (MFI) of mBBR in Human skeletal muscle cells (left panel) and the average mBBr MFI (right panel) from three experiments (n=3). (F) LDH release detection assay for cell death with 1mM FeCl_3_ for 24h, quantified as 490nm absorbance (n=4). (G) Representative EVOS live cell images of apoptotic cells with compromised plasma membrane detected by Image-iT DEAD kit, presented as % of total nuclei and (H) its quantification (n=3). Scale bar denotes 10µm for A, C and 50µm for G. All graphs show mean ± SEM and * = P<0.05, ** = P<0.01, *** = P<0.001 versus control.

**Supplementary Figure 2. Quantification of eGFP-LC3II fluoresce by flow cytometry.**

(A) Gating strategy showing eGFP fluorescence in wildtype (WT) L6 and LC3B-eGFP-mCherry L6 cells. (B) Representative flow cytometry histograms of eGFP fluorescence in eGFP-mCherry-LC3B L6 cells before (blue) and after 0.05% saponin treatment (red). WT L6 cells served as a baseline control. Mean fluorescence intensities (dMFI) were quantified by subtracting the MFI of WT L6 cells from that of eGFP-mCherry-LC3B cells (C) and representative confocal microscopy image of LC3B-eGFP-mCherry L6 cells washed with/without 0.05% saponin. (D) Representative flow cytometry histograms showing the mean fluorescence intensity (MFI) of eGFP in LC3B-eGFP-mCherry L6 cells following treatment with 500nM Rapamycin (top panel) or 30μM CQ (bottom panel) for 1, 2, 4 or 24h of before (left panel) and after 0.05% saponin treatment (right panel).
