# Supplementary material for: Autophagy deficiency exacerbates iron overload induced reactive oxygen species production and apoptotic cell death in skeletal muscle cells
Source: Cell Death Dis. 2023 Apr 7;14(4):252. doi: 10.1038/s41419-022-05484-3 (PMC10081999; doi:10.1038/s41419-022-05484-3)
Supplement: Supplementary file 4 — Original Data File [file 41419_2022_5484_MOESM4_ESM.pptx]

## Slide 1
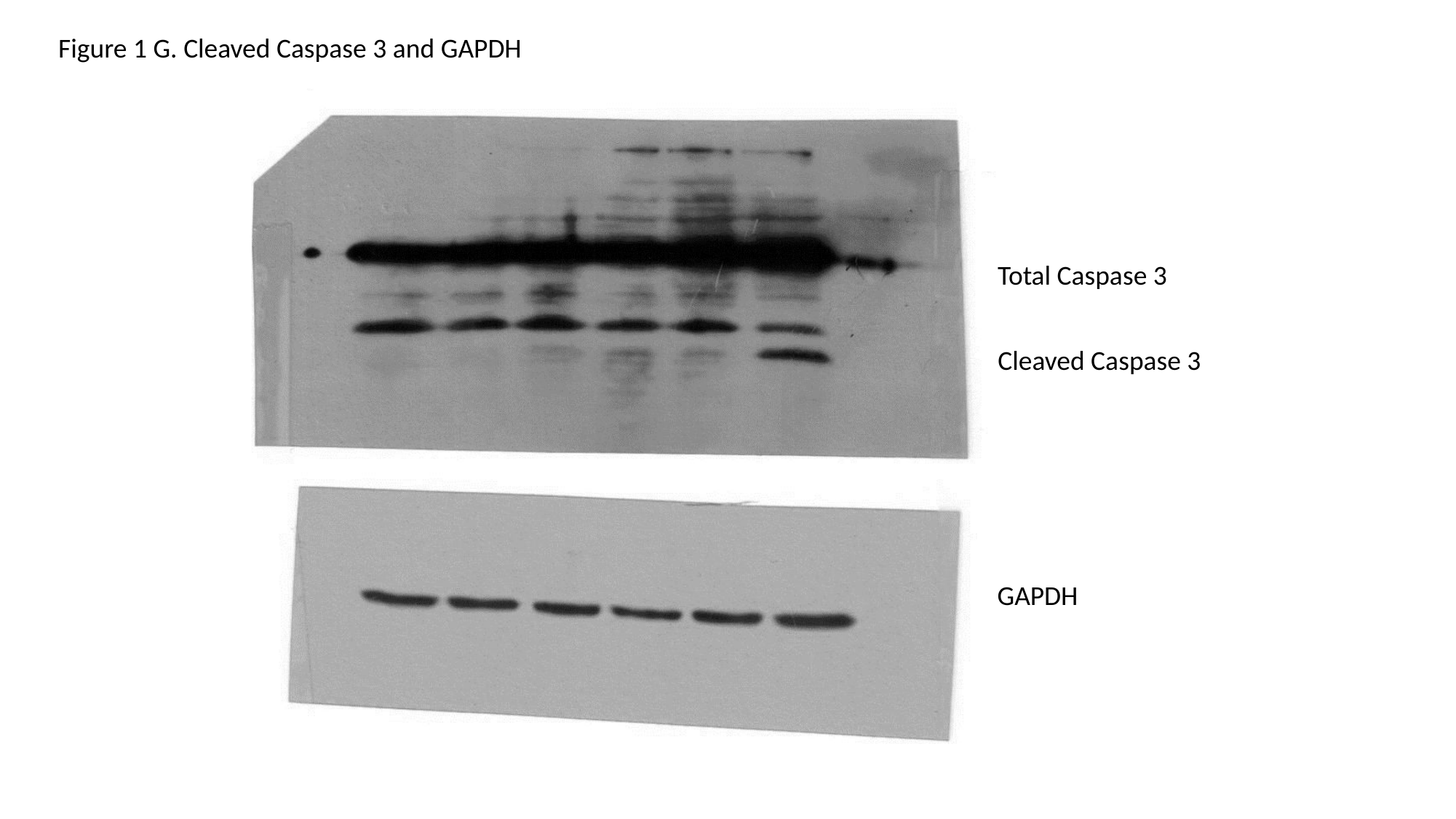

Figure 1 G. Cleaved Caspase 3 and GAPDH
Total Caspase 3
Cleaved Caspase 3
GAPDH

## Slide 2
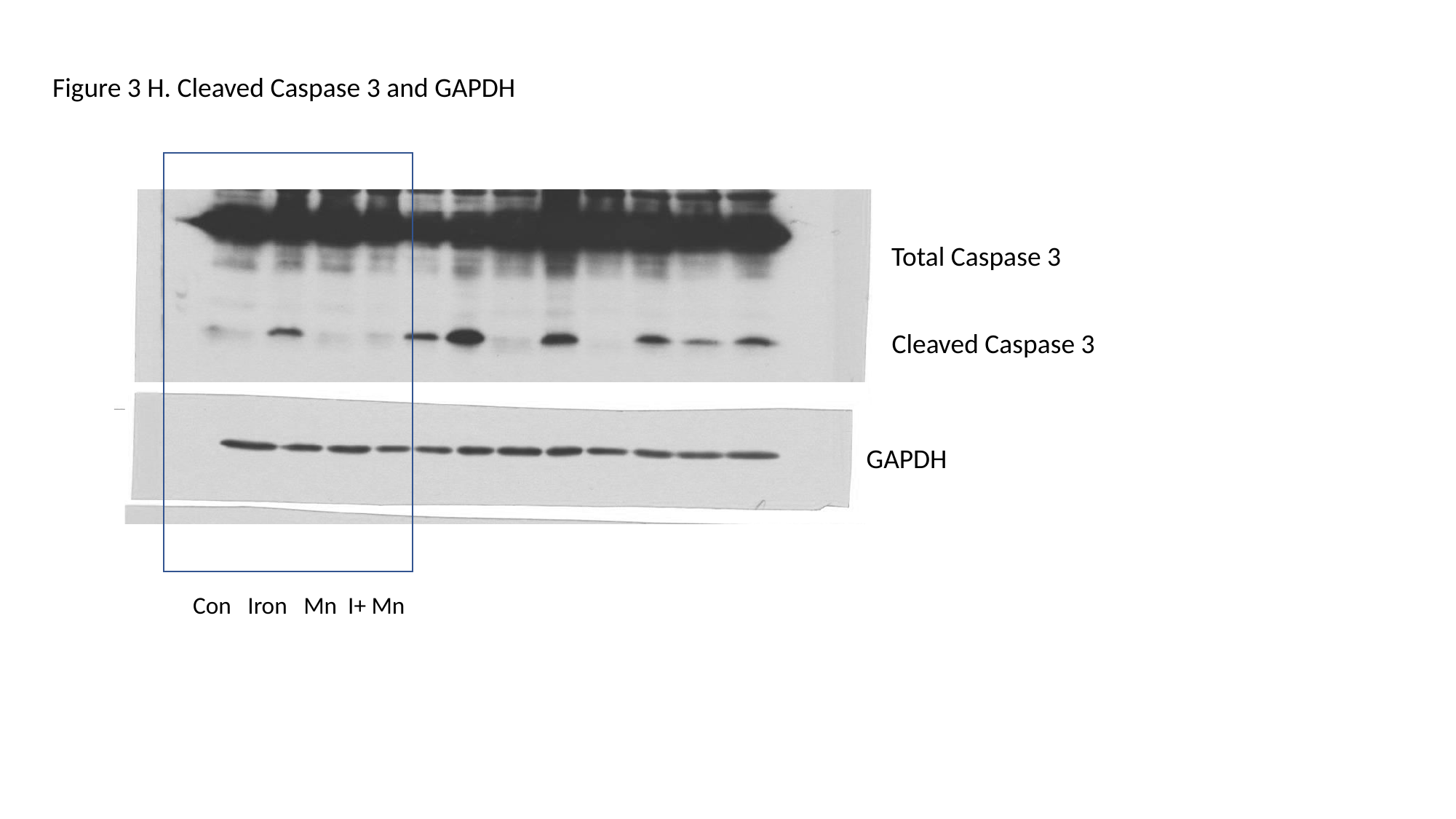

Figure 3 H. Cleaved Caspase 3 and GAPDH
Total Caspase 3
Cleaved Caspase 3
GAPDH
Con Iron Mn I+ Mn

## Slide 3
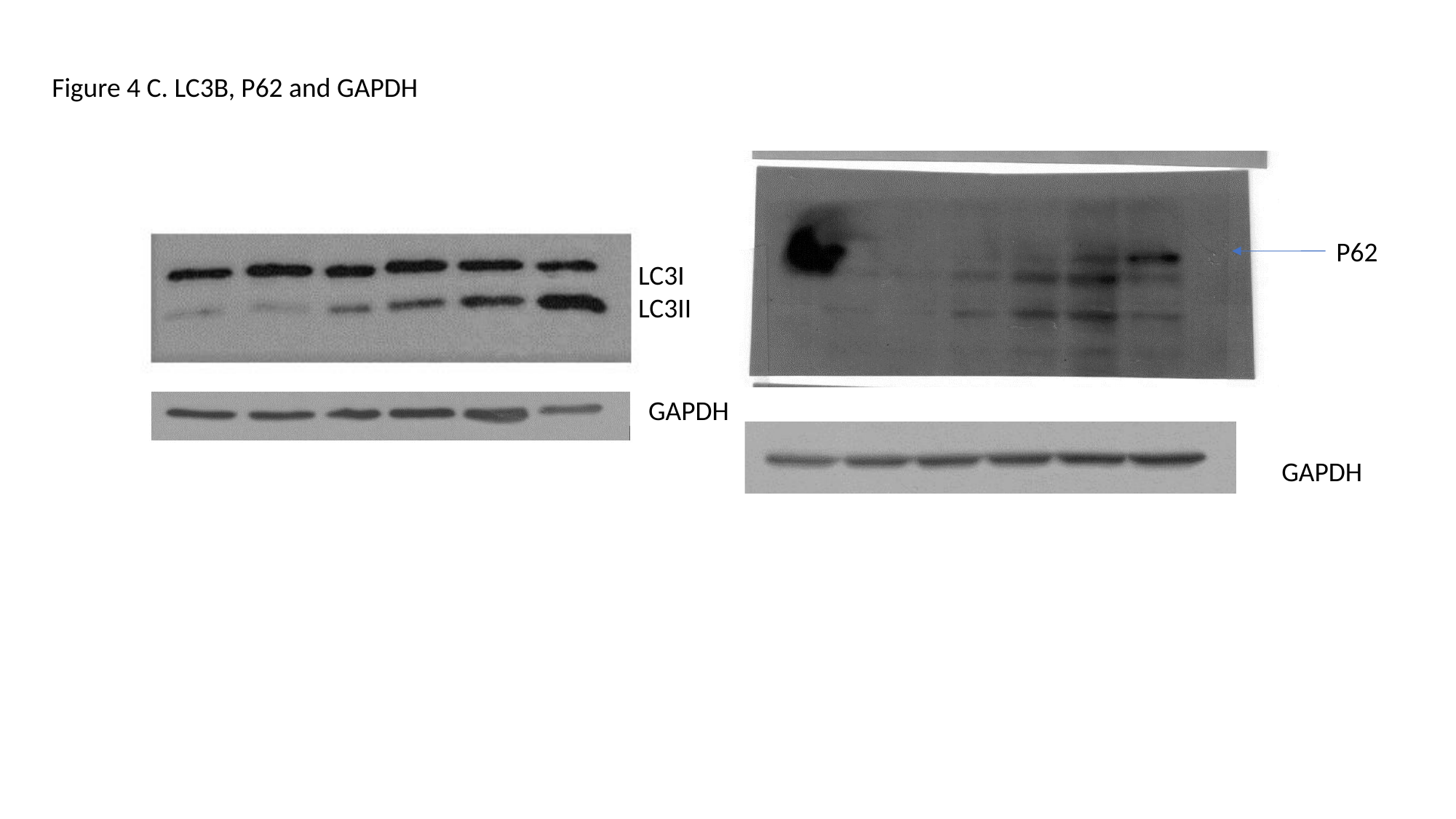

Figure 4 C. LC3B, P62 and GAPDH
P62
LC3I
LC3II
GAPDH
GAPDH
